# Development of a cancer metastasis-on-chip assay for high throughput drug screening

**DOI:** 10.3389/fonc.2023.1269376

**Published:** 2024-01-04

**Authors:** Lutfiye Yildiz Ozer, Hend Salah Fayed, Johan Ericsson, Ayman Al Haj Zen

**Affiliations:** College of Health and Life Sciences, Hamad bin Khalifa University, Doha, Qatar

**Keywords:** cancer metastasis, intravasation, organ-on-a chip, high content screening, targeted anti-cancer therapy

## Abstract

Metastasis is the cause of most triple-negative breast cancer deaths, yet anti-metastatic therapeutics remain limited. To develop new therapeutics to prevent metastasis, pathophysiologically relevant assays that recapitulate tumor microenvironment is essential for disease modeling and drug discovery. Here, we have developed a microfluidic metastasis-on-chip assay of the early stages of cancer metastasis integrated with the triple-negative breast cancer cell line (MDA-MB-231), stromal fibroblasts and a perfused microvessel. High-content imaging with automated quantification methods was optimized to assess the tumor cell invasion and intravasation within the model. Cell invasion and intravasation were enhanced when fibroblasts co-cultured with a breast cancer cell line (MDA-MB-231). However, the non-invasive breast cancer cell line, MCF7, remained non-invasive in our model, even in the presence of fibroblasts. High-content screening of a targeted anti-cancer therapy drug library was conducted to evaluate the drug response sensitivity of the optimized model. Through this screening, we identified 30 compounds that reduced the tumor intravasation by 60% compared to controls. Multi-parametric phenotypic analysis was applied by combining the data from the metastasis-on-chip, cell proliferation and 2D cell migration screens, revealing that the drug library was clustered into eight distinct groups with similar drug responses. Notably, MEK inhibitors were enriched in cluster cell invasion and intravasation. In contrast, drugs with molecular targets: ABL, KIT, PDGF, SRC, and VEGFR were enriched in the drug clusters showing a strong effect on tumor cell intravasation with less impact on cell invasion or cell proliferation, of which, Imatinib, a multi-kinase inhibitor targeting BCR-ABL/PDGFR/KIT. Further experimental analysis showed that Imatinib enhanced endothelial barrier stability as measured by trans-endothelial electrical resistance and significantly reduced the trans-endothelial invasion activity of tumor cells. Our findings demonstrate the potential of our metastasis-on-chip assay as a powerful tool for studying cancer metastasis biology, drug discovery aims, and assessing drug responses, offering prospects for personalized anti-metastatic therapies for triple-negative breast cancer patients.

## Introduction

Metastasis is the hallmark of cancer progression and constitutes the primary cause of death for >90% of patients with cancer (1). In the case of breast cancer, about a third of advanced metastatic breast cancer is initially diagnosed as early-stage disease before progressing to metastatic disease (2). Thus, preventing metastasis progression by more effective early-stage breast cancer therapeutic intervention is an important strategy to improve patient survival. For instance, adjuvant anti-cancer systemic therapy is offered after surgical resection to patients with early triple-negative breast cancer, an aggressive subtype of breast cancer with a high risk of recurrence and metastasis (3). However, therapeutics targeting the cancer progression to metastatic disease remain limited.

One major obstacle that might slow the development of new anti-metastatic cancer therapies is the lack of pre-clinical disease models for drug screening that recapitulate the complexity of cancer metastasis, including host-stroma–tumor cell interactions (4, 5). The process of metastasis results from a cascade of events that allow cancer cells to escape from the tumor primary site, intravasate into circulation, survive in the circulatory system, extravasate, and grow at distant locations in the body (6). The different components of tumor microenvironment (TME) create specific patterns of concentration gradients in space and time that contribute to tumor cell differentiation, migration, invasion and intravasation (7). In addition to tumor cells, TME comprises stromal fibroblasts, endothelial cells, adipocytes, and immune cells, such as macrophages and lymphocytes and the extracellular matrix components, such as collagen and fibronectin (8). For instance, stromal fibroblasts stimulate survival and proliferation signaling pathways through heterotypic signaling with cancer cells (9). Cancer-associated fibroblasts can reshape the tumor stromal extracellular matrix by either enhancing the accumulation of its constituents, secreting degrading enzymes, or remodeling the extracellular matrix (10). The invasive tumor cells interact with the microvasculature, since it is the primary route for disseminating tumor cells in cancer (11). Intravasation is the critical step in cancer metastasis, during which tumor cells transmigrate the vascular wall and enter the bloodstream (12). Therefore, it is essential that *in vitro* modeling of cancer metastasis reflects the complexity of tumor microenvironment which would enhance the sensitivity of the model to capture drug response. Three-dimensional (3D) tumor cell models were developed to recreate its cellular and extracellular microenvironment (13, 14) which lacks in two-dimensional (2D) cultures. For instance, 3D spheroids and organoids cellular models can recapitulate the *in vivo* tumor microenvironment including tumor hypoxia (15, 16). The Boyden chamber model, which consists of two compartments divided by a porous membrane, enables the creation of a 3D hydrogel environment with the possibility to co-culture different cell types (17). It was widely utilized to evaluate trans-endothelial migration of tumor cells (18, 19), and tumor cell invasiveness (20). Nevertheless, to mimic the cancer metastasis, the 3D models display the lack of vasculature, perfusion, and fluid shear stress (21). Here, microfluidic organ-on-chip systems (22, 23), allows unique features over the conventional 3D culture technologies since it is their ability to integrate compartments that can be used to mimic the intra-tumoral microvessels (24). In addition, the constructed microvessels can be perfused, recapitulating the hemodynamic force generated by blood flow, which is essential in establishing an equivalent *in vivo* physiological function of capillaries (25). Several microfluidic platforms have been developed to recapitulate some steps of the metastasis cascade for breast cancer and evaluate the cell-cell interactions (26). For instance, Zervantonakis et al. recreated the tumor-vascular interface using an organ-on-a-chip device, showing that TNF-α-mediated macrophages impair the endothelial barrier (24). Another study described the development of an organ-on-a-chip device in which a breast cancer cell line was cultured into different 3D hydrogel-based matrices positioned side-by-side. This model evaluated 3D chemotactic invasion in response to Epidermal Growth Factor (EGF) (27). A recent microfluidics model has been developed to mimic the mammary ducts by incorporating MCF7 cells that form ducts with lumen surrounded by collagen and co-cultured with fibroblasts. This device allowed the investigation of stromal cell effects on the estrogenic response of MCF7 cells (28). However, remaining technological challenges must be overcome to apply organ-on-a-chip cultures to automated high-throughput phenotypic drug discovery.

To address these challenges, in the present study, we employed the OrganoPlate^®^ platform (29, 30), to develop a high throughput microfluidic organ-on-chip assay that mimics the early stages of cancer metastasis, including tumor cell invasion and intravasation, and performed a drug profiling screen of an annotated targeted anti-cancer drug library to validate the suitability of our optimized assay as a pre-clinical drug testing and screening platform. An *in vitro* model to assess metastasis and drug sensitivity could provide an excellent opportunity to enhance our understanding of tumor metastasis and apply it to precision medicine.

## Materials and methods

### Cell culture

Pooled human umbilical vein endothelial cells (HUVECs) were purchased from Thermo Fisher Scientific. HUVECs were maintained in an Endothelial Growth Medium (EGM-2, Lonza) containing 2% fetal bovine serum (FBS). Normal adult dermal fibroblasts (Thermo Fisher Scientific) were grown in a human fibroblast expansion basal medium supplemented with a low serum growth supplement (Thermo Fisher Scientific). All primary cells were used between passages 4 and 8. Two adenocarcinoma breast cancer cell lines: MCF7 and MDA-MB-231, were obtained from the ATCC. Both cell lines were grown in DMEM media with 10% FBS and 1% Penicillin-Streptomycin mixture. All cell cultures were maintained in an incubator with 37°C/5% CO_2_. We used Accutase^®^ solution (Sigma) for all cell expansion procedures.

### The compound library

The targeted anti-cancer therapy drug library (HY-L080) was purchased from MedChemExpress and comprised 99 targeted therapy drugs used in targeted cancer therapy. The compounds were arrayed in 96-well plates at a concentration of 10 mM in DMSO solution. Compounds were diluted to obtain a final concentration of 100 μM in an intermediate 96-well plate. The compounds were diluted in EGM-2 media to a final concentration of 5 μM for screen use. DMSO 0.05% solution was used as a vehicle.

### GFP-labelling of tumor cells

The GFP was expressed at the whole cell level, allowing the tumor cells to be labeled for cell-tracking studies. Both tumor cell lines: MCF7 and MDA-MB-231, stably expressed GFP by transduction of a pre-made eGFP-puro-CMV-lentiviral vector (GeneCopoeia). Cell lines were incubated with puromycin every 3–4 days until drug-resistant colonies became dominant.

### Metastasis-on-a chip assay

To establish a perfused microvessel in culture, we used the OrganoPlate^®^-3-lane (Mimetas), a microfluidic organ-on-chip platform established on a 384-well plate format with 40 or 64 microfluidic chips. Briefly, 2.2 μl of type I collagen with a final concentration of 5 mg/ml (stock solution of 10 mg/ml rat tail type I collagen (Corning) was neutralized with 10% 37 g/l Na_2_CO_3_ (pH 9.5) and 10% 1 M HEPES buffer) and was loaded in the middle channel of the chip. The plate was incubated for 15 min at 37°C, 5% CO_2_, until the polymerization of collagen gel. Next, 40 μl of fibronectin in PBS with a final concentration of 10 μg/ml was added into the inlets of both the perfusion channels. The next day, endothelial cells were resuspended in EGM-2 media at 10,000 cells/μl. After the washing step with PBS, 50 μl of the EGM-2 media was added to the outlet of perfusion channels. Next, 2 μl of cell suspension was dispensed into the inlet of one of the perfusion channels (microvessel perfusion channel). The plate was then tilted for 2-3 hours in a humidified incubator before adding 50 μl of EGM-2 media to the perfusion inlet wells. Plates were moved to the Mimetas rocker platform in the cell culture incubator, where they were exposed to fluid shear stress induced by a bi-directional pulsatile flow generated by leveling (31). The leveling was set at a 14° angle and 8 min interval, corresponding to a pulsatile flow with 2.5 dyne/cm^2^. These settings were maintained during the experiment period. The EGM-2 growth media was changed every 48 hours. OrganoPlates^®^ were incubated for 4 days until a monolayer endothelial tube (microvessel) formed. A mixture of cell suspension of GFP-labelled cancer cell line (4x10^6^ cells per ml) and adult dermal fibroblasts (4x10^6^ cells per ml) were prepared in DMEM media. 3 μl of cell suspension mixture was added into the inlet of the opposite perfusion channel (tumor cell channel). OrganoPlates were then incubated for 2-3 hours at 37°C, 5% CO_2_ until complete cell attachment. Then, 50 μl of DMEM growth media was added into the inlet and outlet of tumor perfusion channel. Plates were then moved to the Mimetas rocker platform in the cell culture incubator. HUVEC grown in the microvessel perfusion channel were maintained using the EGM2 media, whereas the MDA-MB-231/fibroblasts grown in the tumor perfusion channel were maintained using DMEM media.

### Drug screen

Drug screening was conducted using the optimized metastasis-on-chip assay. One day after tumor cells/fibroblasts seeding, compounds were added at a final concentration of 5 μM into the inlet and outlet microvessel perfusion channels. The drugs were replenished once after three days. According to the manufacturer’s recommendation, cell culture in the OrganoPlate^®^ requires medium culture refreshment every 2-3 days. Besides, several previous studies that used OrganoPlates^®^, the media refreshment interval ranged from two days up to six days. Six days after tumor cell seeding, the chips were fixed and stained for cell imaging.

### Cell staining and immunostaining

All cell staining procedures in OrganoPlate^®^ were performed per the manufacturer’s instructions (32). Briefly, OrganoPlates^®^ were fixed using 4% paraformaldehyde in PBS for 15 min and washed three times with PBS for 5 min. OrganoPlates^®^ were incubated with blocking and permeabilization buffer containing 3% Bovine Serum Albumin and TWEEN 0.03% in PBS for 30 min. Next, the chips were incubated with the primary antibody, mouse anti-human VE-Cadherin (1:30, Santa Cruz) overnight at 4°C. The chips were washed twice for 5 min with PBS and then incubated with the secondary antibody: donkey anti-mouse Alexa Fluor 568 (Thermo Fisher Scientific) for 1 hour at room temperature. Hoechst 33342 (Thermo Fisher Scientific) was used for nuclear staining, and Deep Red Cell Mask (Thermo Fisher Scientific) was used to delineate the cell body.

### Image acquisition and analysis

**Figure 1:**
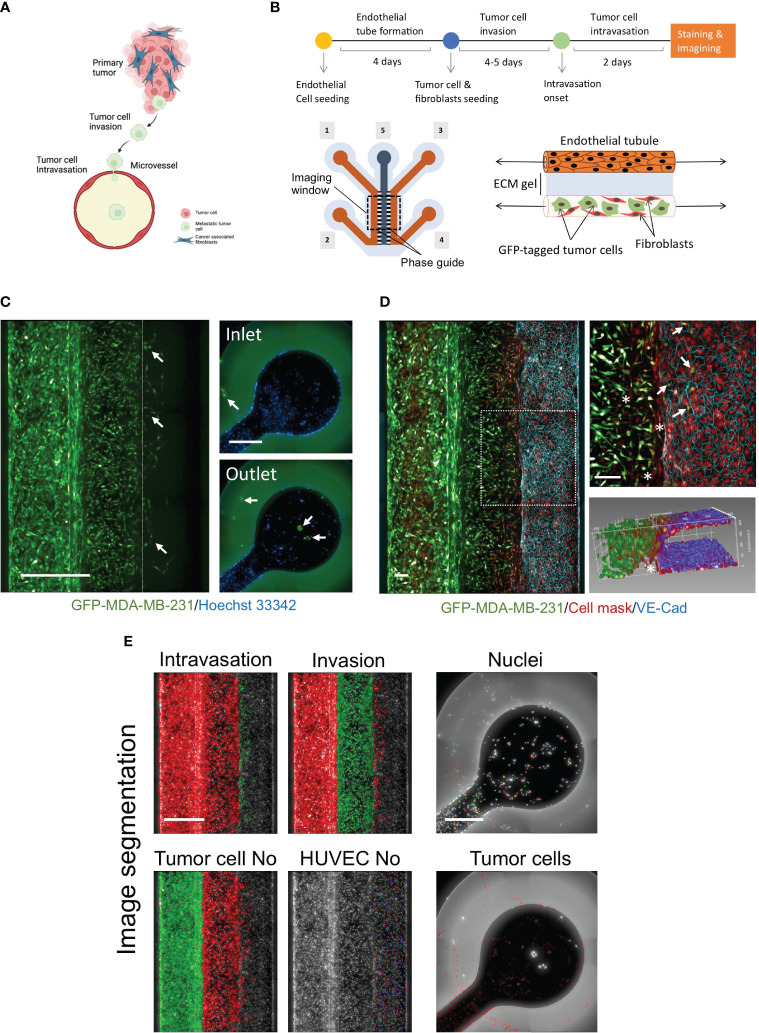
Development of novel a high throughput organ-on-a chip assay to mimic early stages of cancer metastasis. **(A)** Cellular components of tumor microenvironment and early stages of metastasis. Created with BioRender.com. **(B)** OrganoPlates® 3-lane design was used to optimize the metastasis-on-chip model. [1] & [2] tumor channel inlet and outlet. [3] & [4] microvessel perfusion channel inlet and outlet [5] Collagen gel middle channel inlet. *Upper*; Schematic diagram showing the steps of the assay procedure. **(C)** A mixture of GFP-labelled MDA-MB-231 and adult fibroblasts (1:1) were co-cultured with the newly formed microvessel to mimic the tumor cell invasion and intravasation. The dotted line delimits the microvessel border. Intravasated tumor cells circulated within the media flow to reach the inlet and outlet. (arrow) intravasated tumor cells detected in the microvessel and the inlet/outlet. Scale bar = 500 μm. **(D)** a representative confocal z-stack image showing the HUVEC formed a microvessel with continuous perfusion. To assess the endothelium integrity, we stained the endothelial cell junctions using an antibody against VE-cadherin (blue), scale bar = 100 μm. (arrow: intravasated tumor cells). (*: invaded fibroblasts). An 3D reconstruction of the microvessel illustrated tumor cells intravasated the microvessel. **(E)** The image quantification process of metastasis-on-chip assay. The segmentation of endothelial cells and the tumor cell populations: invaded tumor cells, intravasated tumor cells, and tumor cells in the perfusion channels. (*Green mask*) selected tumor cell population of interest. (*Red mask*) discarded tumor cell populations. The number of all selected cell populations was quantified using Harmony® automated image analysis software. Scale bar = 500 μm.

OrganoPlates^®^ were imaged with a 10× objective using the CLS operetta high content microscopy system (PerkinElmer). Images of chip channels were taken under spinning disk confocal mode with 40 z-steps with 5 µm spacing. Four adjacent view fields were acquired to cover chip channels, and four others were acquired to cover the microvessel perfusion inlet and outlet. The chip confocal image tiles were stacked and stitched to perform the image segmentation and quantification using the built-in Harmony^®^ image analysis software (**Figure 1E**). For screen data analysis, all quantified data were normalized to the negative controls of each plate.

### Live cell proliferation screen

GFP-labelled MDA-MB-231 cells were seeded at a density of 5000 cells in 96-well format plates. The next day, cells were incubated with the compound library at a final concentration of 5 μM for 48 hours. We used DMSO (vehicle) and 5 μM Sunitinib (TOCRIS) as negative and positive controls, respectively. The screen was conducted in duplicate. Screen plates were imaged immediately (t0), 24 h, and 48 h after compound addition using a controlled environment chamber of the CLS Operetta high content imaging system (PerkinElmer). Nine view fields were acquired for each well using a 10x objective. Based on GFP cell labelling, cell number was quantified using Harmony^®^ image analysis software. The duplicate data were averaged and then normalized to their respective controls at 0h (**Supplementary Figure 2**). Next, each drug’s data was further normalized to the mean value of the vehicle (DMSO) negative control of the respective plate.

### Tumor cell 2D migration screen

GFP-labelled MDA-MB-231 cells were seeded at 18,000 cells per well in 96-well plates fitted with stoppers (Oris™ Cell Migration Assay, Platypus Technologies). Cells were incubated overnight at 37°C and 5% CO_2_ before removing the stoppers. After removing the stoppers, the library compounds were added at a final concentration of 5 μM for 48 h. DMSO (vehicle) and 5 μM ROCK inhibitor (GSK429286, TOCRIS) were used as negative and positive controls, respectively. The screen was conducted in duplicate. The plates were imaged at hour 0 and hour 48 upon removal of stoppers using the CLS Operetta high content imaging system (PerkinElmer). Nine view fields were acquired for each well using a 10x objective. The view field image tiles were stitched into one image for quantification. The number of migrated cells into the gap was quantified using Harmony^®^ image analysis software and NIH Image J software (**Supplementary Figure 3**). Next, each compound’s data value was normalized to the mean value of the vehicle (DMSO) negative control of the respective plate.

### Multiparametric analysis

We employed the uniform manifold approximation and projection (UMAP) method to visualize and characterize clusters of drug treatments based on the following normalized phenotypic parameters: intravasation, invasion, proliferation, migration, endothelial cell number, and tumor cell number in tumor channels. UMAP-based dimension reduction to two dimensions was carried out in R-studio using the open-source ‘umap’ package (https://cran.r-project.org/web/packages/umap/vignettes/umap.html) (33). Following uploading the dataset into R-studio, we tuned several parameters from the default settings of the ready-to-use algorithm to determine optimal settings. We found that selecting a low value for the minimum distance as 0.1 and the number of neighbors as 6 generated clear separations between clusters. Both parameters are related to two matrices: one matrix links each data point to a fixed number of nearest neighbors, and the second matrix determines the distances between the neighboring data points. The UMAP results were then plotted in Prism GraphPad version 9. Next, the normalized phenotypic parameters of drugs in each cluster were averaged to create an illustrative heatmap for the cluster phenotypic features.

### Trans-endothelial electrical resistance assay

To evaluate the integrity of endothelial cell monolayer, trans-endothelial electrical resistance (TEER) was measured for HUVEC seeded on fibronectin-coated 24-well transparent transwell inserts with a 0.4 µm pore size, as previously described (34). The Millicell^®^ ERS (Electrical Resistance System, Merck) was used to measure total resistance (Ω). HUVEC were seeded at a density of (5 × 10^4^ cells) into the insert (upper chamber), and 1 ml of medium per well was added in the lower chamber. Cells were grown until confluency, confirmed by observing the cobblestone-like appearance using phase contrast microscopy (4 days after seeding). A mixture of MDA-MB-231/fibroblasts (5 × 10^4^ cells for each cell type) was seeded in the bottom chamber. Two days later, the electrical resistance was measured (in triplicate) per well. The mean value was then used for the final data calculation. The values obtained from a blank insert (no cells) were subtracted to calculate TEER to give the net resistance. The resulting value was normalized by multiplying it by the surface area of the insert (0.32 mm^2^).

### Trans-endothelial tumor invasion coculture assay

HUVEC were seeded in the perfusion inlet of the OrganoPlate^®^-3-lane (Mimetas) to form an endothelial tube as described above. Cells were maintained in the EGM-2 media for four days until the formation of a monolayer endothelial tube. At day 5, EGM-2 media was removed, and Imatinib (5 μM) or Vehicle (DMSO) was added and incubated for 2 hours at 37°C with 5% CO_2_. 50 µL of GFP-labelled MDA-MB-231 cell suspension with a density of 100,000 cells per ml was added into the inlet and outlet of the microvessel perfusion channel, followed by incubation for 30 minutes on the Mimetas rocker platform. The chips were washed with EGM-2 media, and then fresh EGM-2 media with Imatinib (5 μM) or Vehicle (DMSO) were added to the inlet and outlet of the microvessel perfusion channel. The OrganoPlate^®^ was then incubated at 37°C with 5% CO_2_ on a rocking platform for three days. Next, cells were fixed and stained with anti-VE-cadherin and Hoechst 33342.

### Statistical analysis

Statistics were carried out using Prism GraphPad version 9. Two treatment groups were compared by unpaired t-test. Multiple group comparisons were analyzed by one-way or two-way analysis of variance (ANOVA) with *post hoc* Bonferroni’s test. Three to six independent experiments were performed to guarantee the reproducibility of the findings. To determine the quality and robustness of the high throughput functional assays, we will calculate their robust Z’ factor statistical parameter (35) using positive and negative controls. We applied Fisher’s exact test to determine whether a molecular target was selectively enriched in a drug cluster. A p-value of < 0.05 was considered to be statistically significant.

## Results

### Modelling cancer metastasis using organ-on-a-chip plates

To model cancer metastasis with TME pathophysiologic relevance (**Figure 1A**), we used OrganoPlate^®^ 3-lane plate from Mimetas, a high-throughput platform containing 64 individual chips. This microfluidics system allows continuous perfusion of nutrient-rich media at a shear stress of around 2.5 dyne/cm^2^, sufficient to mimic the microcirculation of capillaries at tumor intravasation sites (36). In this chip design, when the middle channel was dispensed with type I collagen gel via its inlet, two lateral channels were formed due to the phase guide-controlled capillary forces (37). The two compartments simulated the extracellular matrix and blood vessel compartments surrounding tumors (**Figure 1B**). The collagen gel represents the extracellular matrix (ECM) of tumor fibrotic stroma. We created a microvessel by seeding HUVEC in one of the formed perfusion channels coated by fibronectin. Stromal fibroblasts play a critical role in the TME, influencing tumor invasiveness and metastasis, and are the most abundant cell component of TME (38). Thus, we seeded a mixture of adult stromal fibroblasts and GFP-labelled MDA-MB-231, an aggressive metastatic breast cancer cell line in the opposite perfusion channel following the formation of complete endothelial monolayer tube (microvessel). We found that the mixture of adult fibroblasts and MDA-MB-231 with a (1:1) ratio produced the maximal response of MDA-MB-231 for both cell invasion and intravasation process (**Supplementary Figure 1**). Over five days, GFP-labelled tumor cells invaded the ECM gel along with stromal fibroblasts toward the microvessel wall. At day 3 or 4 after tumor cell seeding, several GFP-labelled tumor cells were observed to intravasate across the microvessel wall. Additionally, we could detect the intravasated tumor cells entered the hollow lumen of the microvessel and traveled within the media flow through the channel to adhere to the HUVEC monolayer at the inlet and outlet surface of the microvessel perfusion channel (**Figure 1C**). To confirm the formation of a continuous 3D endothelial barrier, we used the vascular endothelial (VE)-cadherin antibody to visualize the endothelial cell junctions. We found that endothelial cells formed a confluent monolayer covering the whole perfusion channel and the ECM gel-microvessel interface in the presence of invading and intravasating tumor cells. Z-stack images of the ECM gel/microvessel interface were 3D reconstructed to monitor the tumor cells transmigrating the endothelial monolayer (**Figure 1D**, **Supplementary Movie 1**). The chips were imaged to quantify tumor cell intravasation and invasion using the high-content screening system. All nuclei were segmented based on the Hoechst channel. Next, total tumor cells were identified based on their positive GFP fluorescence. The different tumor cell populations were selected based on their position into invaded tumor cells, intravasated tumor cells, and tumor cells in the tumor channel. The endothelial cells of microvessels were also segmented based on their positions in the chip and the absence of cellular GFP fluorescence. The intravasated tumor cells that traveled and seeded in the microvessel perfusion inlets and outlets were identified based on the nuclei staining channel and their positive GFP fluorescence (**Figure 1E**). Thus, four phenotypic parameters were possible to be calculated, including intravasated tumor cell number, invaded tumor cell number, endothelial cell number, and total tumor cell number in the tumor channels.

### Evaluation of the fibroblast role on tumor cell invasion and intravasation

**Figure 2:**
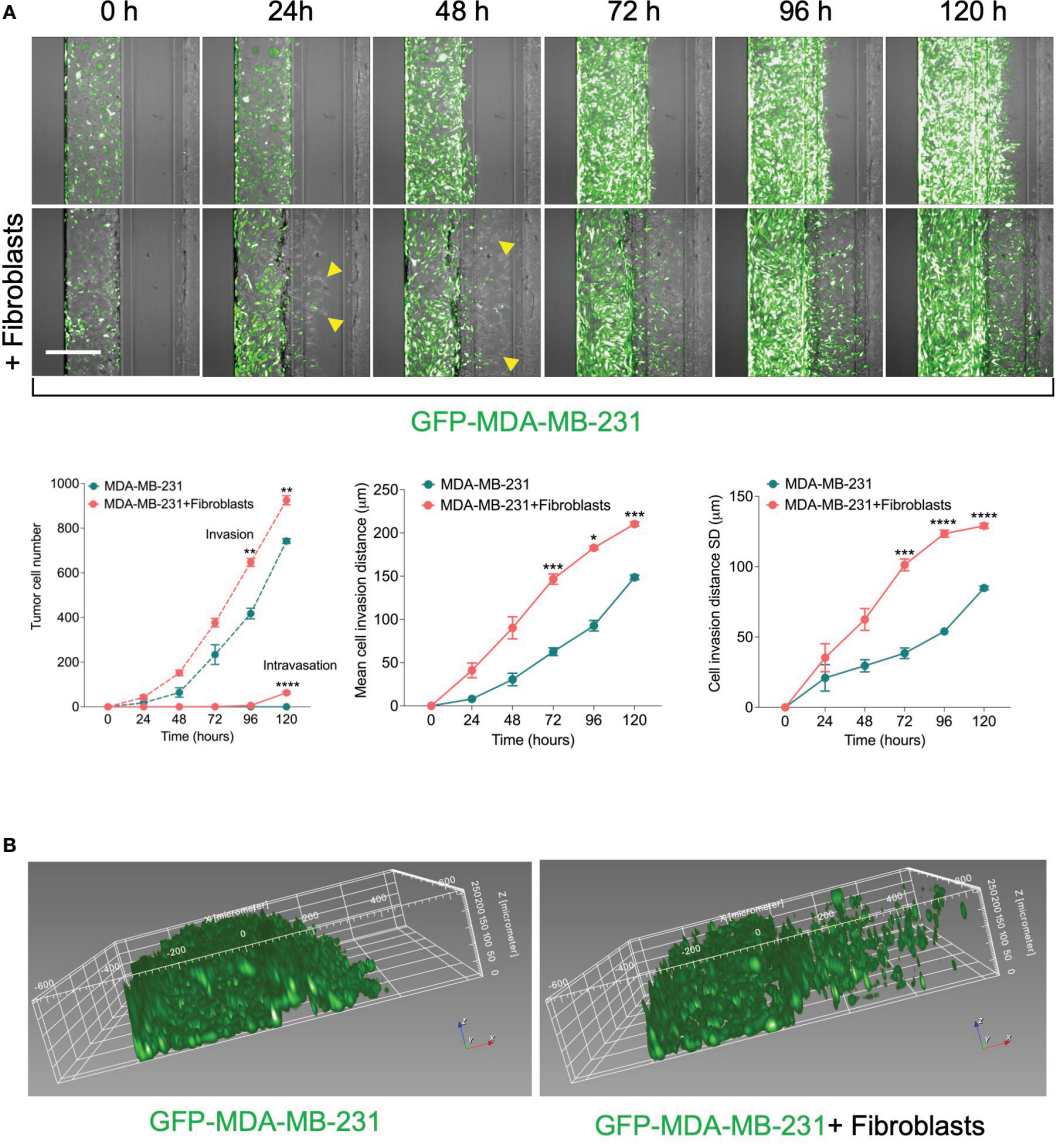
Effect of fibroblasts on the tumor invasion and intravasation. **(A)** Time-course analysis of tumor cell invasion and intravasation in two conditions: 1) GFP-labelled MDA-MB-231 tumor cells were cultured alone. 2) GFP-labelled MDA-MB-231 tumor cells were co-cultured in a mixture with fibroblasts. The chips were imaged daily up to five days (*yellow arrows*: fibroblasts). The invaded and intravasated tumor cells were quantified using Harmony® image analysis software. Scale bar = 500 μm. *Lower*, graphs show the invaded and intravasated MDA-MB-231 cell number, the mean distance of invaded MDA-MB-231 cells, and the distance SD of invaded MDA-MB-231 cells. Data are expressed as mean ± SEM; n=3-4 chips per condition; Two-way ANOVA followed by Bonferroni *post hoc* test, *p<0.05, **p<0.01, ***p<0.001, ****p<0.0001 compared to controls. **(B)** reconstruction showing the spatial position of invaded tumor cells in ECM gel.

**Figure 3:**
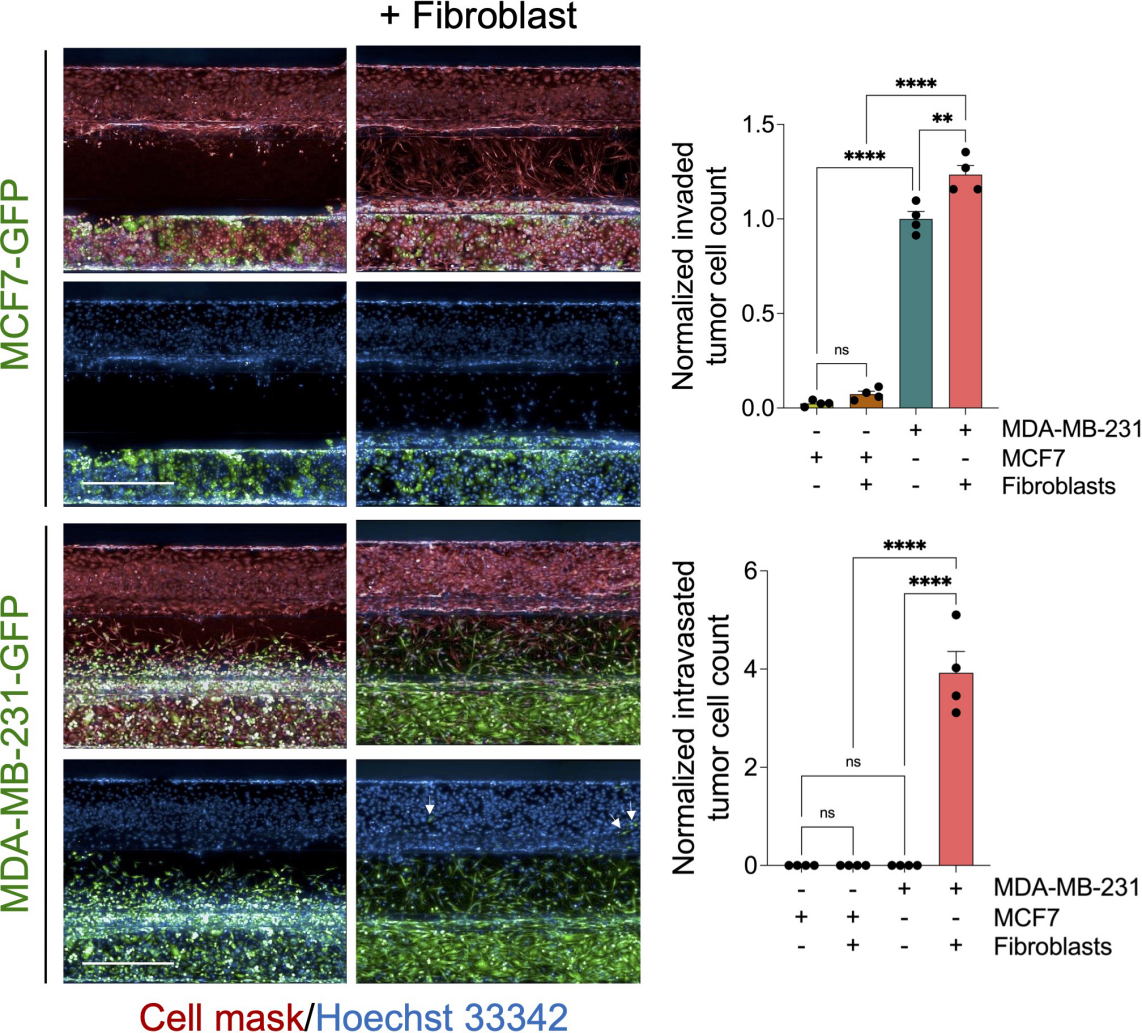
Evaluation of MCF7 tumor cell invasion and intravasation. Representative z-stack confocal images show GFP-labelled MCF7cells (non-invasive breast cancer type) and GFP-labelled MDA-MB-231 cells (invasive breast cancer type) were cultured alone or co-cultured with fibroblast (1:1 ratio) using OrganoPlate®-3 lane design of 40 chips. After five days, cells were fixed and stained with deep red cell mask (red) and Hoechst 33342 (blue). Scale bar = 500 μm. *Right*, graphs showing the normalized invaded and intravasated tumor cell count. Data were normalized to total tumor cell number in the tumor channel. Data were then normalized to the vehicle mean and expressed as mean ± SEM; n = 4 per condition; one-way ANOVA followed by Bonferroni *post hoc* test, **p<0.01 and ****p<0.0001.

To assess the effect in our model, we perform a time course experiment to monitor the interaction between fibroblasts and tumor cells in the metastasis-on-chip assay. When cultured alone or co-cultured with fibroblasts, we found that MDA-MB-231 cells invaded the ECM gel towards the microvessel. Nevertheless, in the case of co-culture with fibroblasts, tumor cell invasion was enhanced as assessed by two parameters: invaded cell number and mean cell invasion distance all over the five days of the assay. The invasion of stromal fibroblasts preceded the tumor cell invasion, indicating that fibroblasts facilitate and lead the tumor cell invasion (**Figure 2A**). MDA-MB-231 invaded the ECM with a collective type of migration. However, when MDA-MB-231 co-cultured with fibroblasts, they invaded the ECM with a single-cell type of migration as visualized by their distribution of invaded tumor cells within the Z-depth of the ECM gel, and by the increase of the SD of cell invasion distance (**Figure 2B**). We detected a few tumor cells (1-3 cells per chip) intravasated the microvessel after three days, reaching 10% of the total invaded cell number at day 5. In contrast, we did not detect any tumor cell intravasation when MDA-MB-231 was cultured alone during this time frame (**Figure 2A**). Notably, the co-cultured fibroblasts were observed to reach the microvessel wall edge on day 2 before MDA-MB-231 cells. However, we did not detect any fibroblasts intravasated inside the microvessel or on the inlet/outlet of the microvessel channel over the experiment period. Next, to assess the model’s selectivity of tumor cells’ metastatic nature, we used the MCF7 breast cell line, which is known as a non-invasive human breast cancer cell line that expressed estrogen and progesterone receptors. After five days of tumor cell seeding, we found that MCF7 remain incapable of invading the ECM gel. The co-culture with fibroblasts did not promote significantly the MCF7 invasion of ECM as compared to MDA-MB-231 (**Figure 3**).

### Phenotypic drug screening using the metastasis-on-a chip model

**Figure 4:**
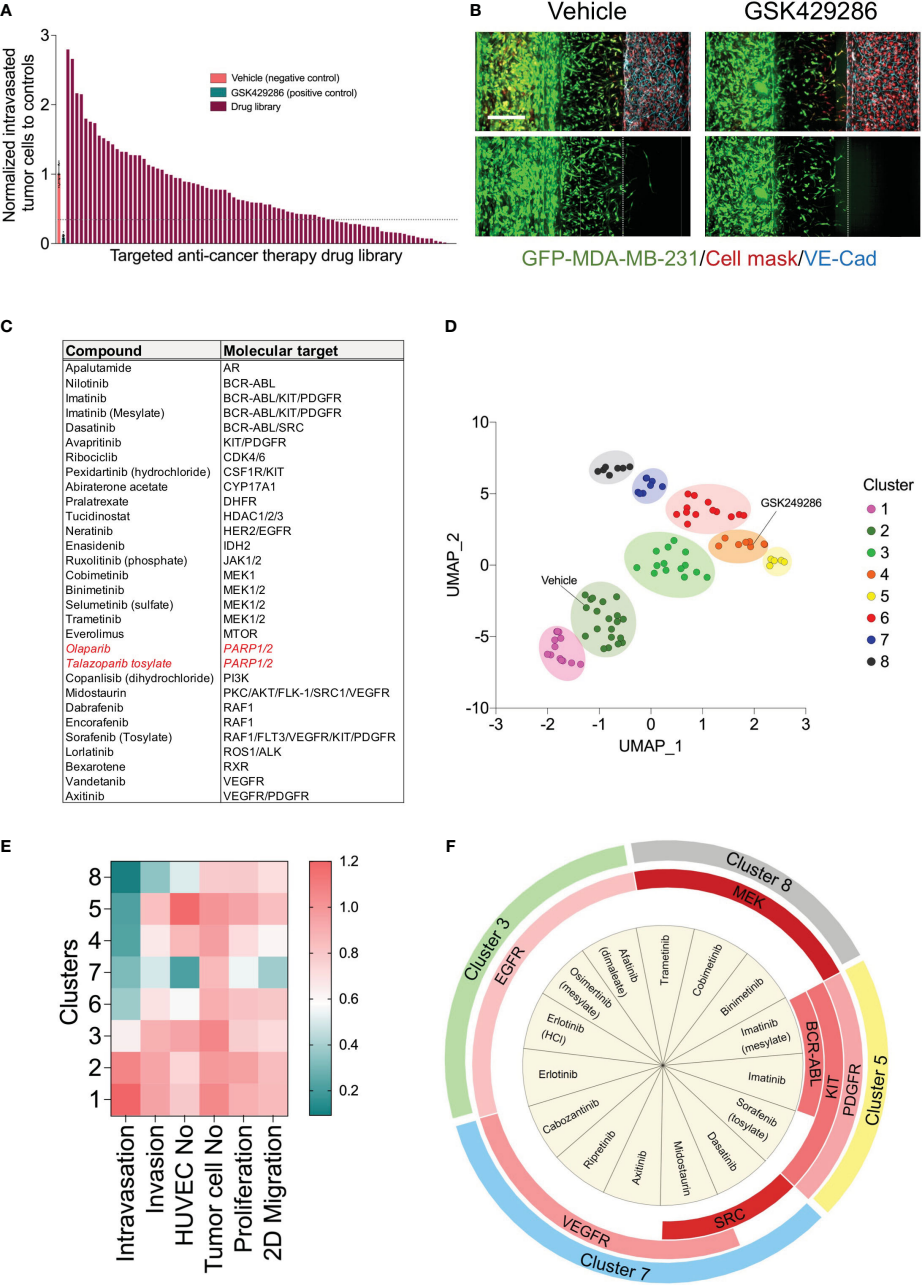
High content drug screen of targeted anti-cancer library to profile their effects on the metastatic behavior of MDA-MB-231 tumor cells. **(A)** Ordered bar chart distribution showing the drug screen data using metastasis-on-chip model. The intravasation and invasion were normalized to controls treated with vehicle (DMSO). Compound inhibitor of tumor intravasation were indicated below the dotted line (hit threshold was calculated as follows: mean_(vehicle)_ - 4x standard deviation_(vehicle)_. Drug library was screen at final concentration of 5 μM. **(B)** Representative confocal Z-stack images for the negative control (DMSO, 0.02%) and the positive control (ROCK inhibitor compound “GSK429286”, 5 μM). Scale bar = 250 μm. **(C)** Compound hit list that reduce cancer breast cell intravasation with their molecular targets. In red, drugs approved clinically as monotherapies for triple-negative breast cancer. **(D)** Uniform manifold approximation (UMAP) analysis comparing phenotypic profile of drug library based upon identified phenotypic groupings. Drugs showed similar phenotypes are gathered in one cluster on the plot. Data point color indicates which cluster each molecule belongs to. **(E)** heatmap showing the phenotypic features of each clusters **(F)** Enrichment analysis of the drug molecular targets was performed by Fisher exact test. Chart showing the molecular targets were significantly enriched in each cluster (p<0.05). Red color intensity is proportional to the significance of Fisher exact test.

To determine whether our metastasis-on-chip model represents a suitable preclinical platform to test drug sensitivity against cancer metastatic activity, we applied our model to a high content screen of a compound library for annotated targeted anti-cancer therapy. First, as part of the screen workflow, we screen the drug library at a final concentration of 5 μM in live tumor cell proliferation assay (**Supplementary Figure 2**) and 2D migration assay (**Supplementary Figure 3**). The drugs that reduced the tumor cell proliferation over 48 hours > 60% were excluded from testing in the subsequent metastasis-on-a chip screen. Since the Rho small GTPases regulate many cellular processes, including cytoskeletal dynamics, cell motility, and cell polarity, which are essential for the metastatic potential of tumor cells (39, 40), and previous studies have proposed the potential of ROCK-targeted therapy for cancer metastasis (41), we used a selective ROCK inhibitor (GSK429286) as positive drug control in the metastasis-on-a chip screen. To confirm the selected ROCK inhibitor efficacy, we evaluated its incubation in the microvessel perfusion or tumor opposite channel on the MDA-MB-231 cell invasiveness. GSK429286 reduced both tumor cell invasion and intravasation when it was added in the microvessel perfusion channel, which would be relevant to the *in vivo* conditions (**Supplementary Figure 4**). Having established that the positive compound targeting ROCK1/2 activity known to be important in metastasis can also reduce MDA-MB-231 invasiveness in our model, we conducted a screen of the selected 86 drugs. HUVEC were seeded in the perfusion channels of the OrganoPlates^®^, in which they formed a microvessel over four days. Subsequently, a mixture of GFP-labelled MDA-MB-231 and fibroblasts with a ratio of 1:1 were added into the opposite channels and incubated for 24 h. The selected library drugs (86 compounds) were then added at a single concentration of 5 μM to the microvessel perfusion channels and incubated for three days. Next, the culture media and drugs were replenished and incubated for additional two days. ROCK1/2 inhibitor (positive control, 5 μM) and DMSO (negative control, 0.05%) were added to each screening plate. The chips were then fixed, stained and imaged using the CLS Operetta high content imaging system. Image data were processed and quantified with the built-in Harmony^®^ image analysis software as described above. Four phenotypic parameters were extracted from the images of each chip: invaded tumor cells, intravasated tumor cells, total tumor cells in the tumor perfusion channels, and the total endothelial cells in the microvessel channels. The invaded and intravasated tumor cell numbers were normalized to the total tumor cell number of the tumor channels for each chip. All parameters were then normalized to the negative control mean (vehicle, DMSO) for each plate. We calculated an average Z′ factor of 0.2 between negative and positive controls. The screen identified 30 drugs inhibiting the MDA-MB-231 cell intravasation < 30% relative to the negative controls, which is equivalent to [mean_vehicle_-4xSD_vehicle_] (**Figures 4A, B**). The identified drugs are targeting BCR-ABL, MEK1/2, RAF1, PDGFR, VEGFR, KIT, and PARP (**Figure 4C**).

**Figure 5:**
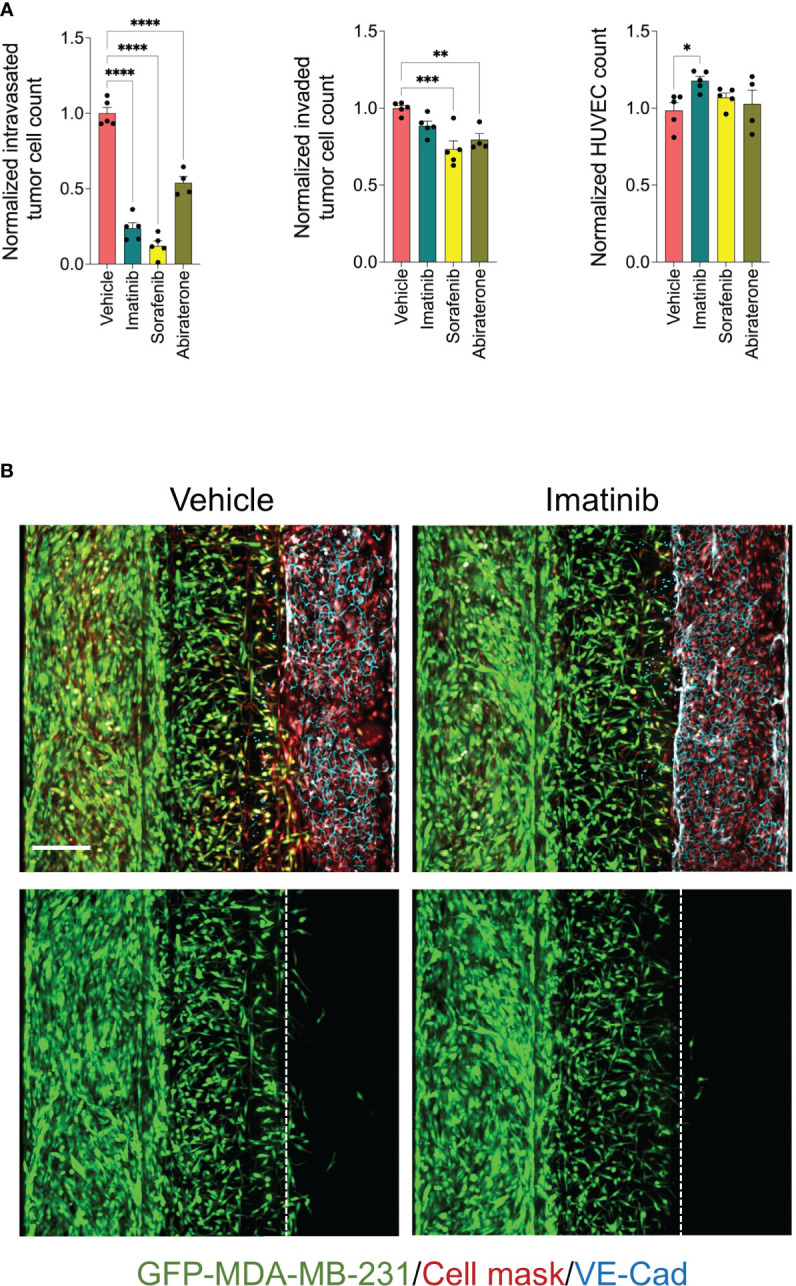
Validation of selected inhibitors of tumor cell intravasation from cluster 5. **(A)** effect of Imatinib, Sorafenib and Abiraterone at a final concentration of 5 μM on tumor cell intravasation, tumor cell invasion, and HUVEC number. Data were normalized to total tumor cell number in the tumor channel. Next, data were normalized to the vehicle mean and expressed as mean ± SEM; n = 4-5 per condition; one-way ANOVA followed by Bonferroni *post hoc* test, *p<0.05, **p<0.01, ***p < 0.001 and ****p<0.0001. **(B)** Representative confocal z-stack images of metastasis-on-chips treated with Imatinib (5 μM) or vehicle (DMSO). Scale bar = 250 μm.

The detected effect of drugs on tumor cell intravasation could also be a secondary effect to other cellular mechanisms of metastatic cascade, such as tumor cell proliferation, motility, and invasion. In addition, we observed that a subset of drug library showed substantial cytotoxicity against endothelium. Therefore, we sought to conduct a clustering analysis to characterize different drug categories based on their phenotypic features resulting from the tumor cell proliferation, 2D migration, and metastasis-on-chip screens. We used UMAP clustering analysis that combined alterations in the following phenotypic descriptors: tumor cell proliferation, tumor cell 2D migration, 3D tumor cell invasion, tumor cell number, tumor cell intravasation, and endothelial cell number. This multi-parametric phenotypic analysis resulted in clustering the drugs into eight distinct groups (**Figure 4D**). The vehicle (negative control) was located in the cluster 2. The influence of compounds in this cluster on tumor cell proliferation and metastatic characteristics was indistinguishable from negative controls. The cluster included many hormone drugs such as Tamoxifen, Fulvestran, and Anastrozole. The compounds in clusters 1 & 3 moderately affected both proliferative and metastatic activity for MDA-MB-231. Indeed, most EGFR and HER2 inhibitors were clustered into clusters 1, 2 & 3. In contrast, compounds in cluster 8 caused the decrease of intravasation, which was associated with a reduction of tumor cell invasion. In this cluster, the reduction of tumor intravasation appeared to be not due to a direct effect on endothelium/tumor cell interaction but somewhat secondary to the cell invasion decrease. The molecular target MEK was enriched in this cluster. We further detected that while the compounds in cluster 7 inhibited the tumor cell intravasation and invasion similarly to the compounds in cluster 8, they also appeared to have a substantial effect on cell proliferation and migration. Both clusters showed substantial effects of endothelial cell toxicity. VEGFR and SRC drug targets were enriched in cluster 8. The compounds in cluster 4 showed reduced metastatic activity, including tumor cell intravasation, invasion, and 2D migration, mildly affecting tumor cell proliferation. ROCK1/2 inhibitor (positive control) was detected in this cluster. In contrast, the compounds in cluster 5 showed around an 80% reduction of tumor cell intravasation with limited effect on tumor cell proliferation, migration, and invasion. Also, these compounds showed a slight increase in the endothelial cell number of the microvessel compared to negative controls (**Figures 4D–F**). Since improving the endothelial barrier function and inhibiting tumor cell-endothelium interactions are the key cellular events to target for preventing cancer metastasis, we focused on cluster 5 for the follow-up studies due to their potential mechanism of action related to endothelium/tumor cell interaction. The molecular targets: BCR/ABL, KIT and PDGFR were enriched in this cluster. We retested three drugs in five biological replicates from this cluster to validate their response. Such compounds were Sorafenib, Imatinib, and Abiraterone acetate. While Sorafenib and Abiraterone significantly reduced both tumor cell intravasation and invasion, Imatinib significantly reduced only the tumor cell intravasation, indicating Imatinib’s anti-metastatic effect could be related to the interaction between tumor cells and endothelium (**Figure 5**). Therefore, we investigated whether Imatinib affects the vascular mechanisms that are involved in cancer metastasis.

### Effect of Imatinib on vascular-mediated mechanisms of cancer metastasis

**Figure 6:**
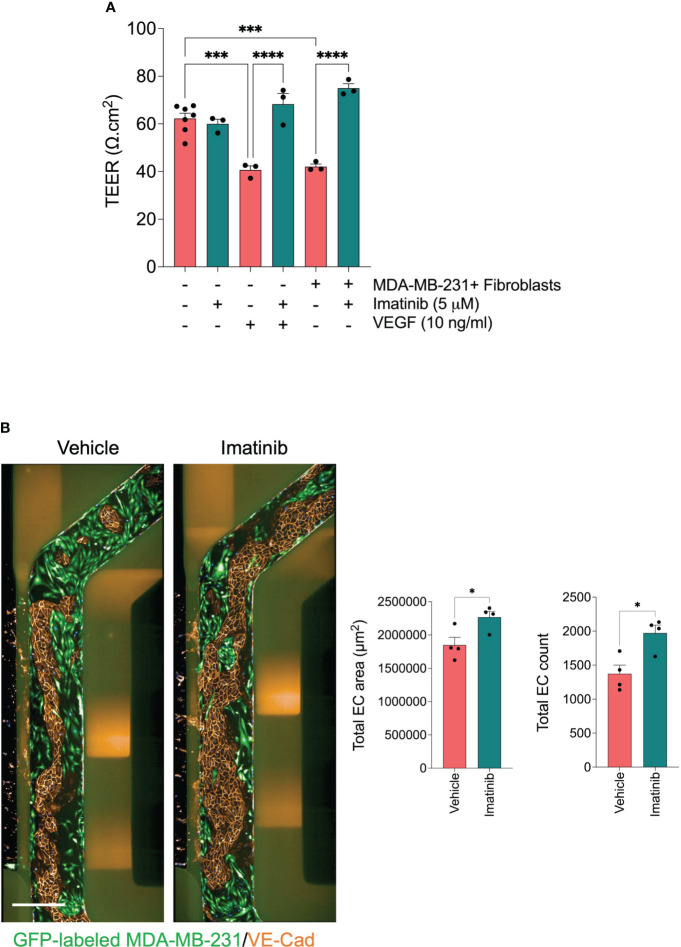
Effect of Imatinib on endothelial barrier function. **(A)** effect of Imatinib on the endothelial monolayer permeability using TEER measurements. Imatinib (5 μM) was incubated in the presence of tumor cell/fibroblast mixture or VEGF 10 ng/ml. TEER values were normalized to blank well and then to the insert surface area. Data are expressed as mean ± SEM; n = 3-6 per condition; one-way ANOVA followed by Bonferroni *post hoc* test, ***p < 0.001 and ****p<0.0001. **(B)** Effect of Imatinib on trans-endothelial tumor invasion. *Left*, representative fluorescent images of microvessel perfusion channels stained with an antibody against VE-Cadherin to visualize the endothelial cells. GFP-labelled MDA-MB-231 cells (green). Scale bar = 500μm. *Right*, Quantification of total endothelial cell number and endothelial cell monolayer area for each condition. Data are expressed as mean ± SEM; n=4 per conditions; unpaired t test, *p < 0.05; compared with vehicle.

Abnormal endothelial barrier function promotes endothelium permeability. Invaded tumor cells tend to intravasate at the sites where endothelial permeability is increased (42). It has been demonstrated in previous studies that Imatinib decreases vascular permeability (43, 44). Since we found that Imatinib reduced the MDA-MB-231 cell intravasation without significant effect of cell invasion, we investigate whether Imatinib can enhance HUVEC monolayer integrity and permeability when they are co-cultured with MDA-MB-231 cells/fibroblasts. To evaluate the effect of Imatinib on endothelial barrier function, we measured transendothelial electrical resistance (TEER) in a secondary assay using the co-culture transwell system. The co-culture of endothelial cell monolayer with the mixture of MDA-MB-231/fibroblasts significantly decreased the endothelial barrier function compared to endothelial cells cultured alone. The co-culture treatment with Imatinib (5 μM) prevents the reduction of endothelial barrier function caused by the co-culture with tumor cells/fibroblasts (**Figure 6A**). VEGF is a potent permeability factor. It increases VE-cadherin phosphorylation leading to its internalization and, subsequently, the loss of the cell-cell contact (45). Confluent HUVEC monolayers were treated with vehicle or VEGF at 5, 10, and 20 ng/ml concentrations. At 48 hours, the TEER measurement showed a significant reduction (20%) from the concentration of 10 ng/ml, compared with controls (**Supplementary Figure 5**). The treatment of confluent HUVEC monolayer with 10 ng/ml of VEGF and 5 μM Imatinib were not significantly different from those of controls, indicating that Imatinib could stabilize HUVEC monolayer and inhibit of VEGF-induced permeability effect (**Figure 6A**). The circulating tumor cells interact with the endothelium, destabilizing the endothelial barrier, which is required to accomplish tumor cell extravasation (12). Therefore, we tested whether Imatinib prevents endothelial cell monolayer disruption by seeding MDA-MB-231 tumor cells into the microvessel perfusion channel with a confluent monolayer of HUVECs in OrganoPlate^®^. Tumor cells adhered to the endothelium and progressively disrupted the endothelial barrier by dislodging endothelial cells and cell expansion over 72 hours, as previously described (46). We found that the endothelial cell number and endothelium area were preserved more in the Imatinib-treated chips than in controls (**Figure 6B**), and many tumor cells remained on the endothelium surface in Imatinib-treated chips. These findings imply that Imatinib reduced the disruption of the endothelial cell monolayer, which is an important effect in preventing the intravasation and extravasation of tumor cells.

## Discussion

Advanced metastatic breast cancer has no cure but can be prevented. One approach is to target the tumor microenvironment to block tumor cell dissemination and eventually form metastatic lesions. Patients with high-risk early-stage cancer who had undergone adjuvant therapy showed a lower recurrence and a prolonged overall survival rate, proving the feasibility of preventing their metastasis-initiating capacity (47). Also, assigning the proper therapeutic intervention can increase the quality of life and longevity. The difference in drug response between various cancer subtypes further reveals the necessity to establish personalized models for patients with pathophysiological relevance, such as microfluidics organ-on-chip technology.

In the current study, we described a novel set-up of a 3D microfluidic organ-on-a-chip assay that mimics the early steps of cancer metastasis. We used the OrganoPlate^®^ platform to develop our assay for its suitability of high throughput screening. Previous studies have used this platform to conduct high throughput analysis for T cell extravasation under flow (48), endothelial cell permeability (31), and the integrity of intestinal tract epithelium (49). The same platform was employed to screen small compound libraries on retinal endothelial permeability (32) and sprouting angiogenesis (50). Our current model incorporates many relevant features of tumor microenvironment, including perfused microvessel, ECM, and stromal fibroblasts. It allows us to visualize and quantify the tumor cell invasion and intravasation, making it suitable for high-throughput and high-content screening. Tumor cell intravasation is controlled by the molecular interactions of invaded tumor cells with the vascular endothelial barrier (51, 52). Dysfunction of the endothelial barrier is a critical factor in the early stages of cancer cell dissemination, and improving the stability of the vascular barrier prevents the progress of metastatic cascade (42, 53). In our study, the presence of tumor cells and fibroblasts was insufficient to induce pro-angiogenic effects on the microvessel in the metastasis-on-chip model. Indeed, the constructed microvessel expressed VE-Cadherin tight junctions and held its tubular integrity over the assay period (6 days). Our data showed that perfused microvessel creates an appropriate gradient to induce directional migration of fibroblasts and tumor cells towards the microvessel wall. Importantly, we demonstrated our model’s accuracy in determining the tumor cells’ aggressivity. MCF7, a poorly aggressive and non-invasive breast cancer cell line, showed no invasive activity in our assay, even when co-cultured with fibroblasts.

Stromal fibroblasts are an essential cell component to be integrated into the *in vitro* models of breast cancer metastasis to recapitulate the *in vivo* tumor microenvironment. Many solid tumors, such as breast cancer, are often fibrotic with dense extracellular matrix (54, 55). During cancer progression, the ability of tumor cells to invade such dense ECM deposition depends on their EMT transition and paracrine effects of cancer-associated cells such as stromal fibroblasts and macrophages to facilitate tumor progression and invasion (56). Stromal fibroblasts contribute significantly to initiating tumor cell dissemination from their primary sites. Many studies showed that the physical contact between fibroblasts and tumor cells is required to align and contract the extracellular matrix to enhance tumor cell invasion (57–59). However, other studies demonstrated that fibroblasts act as leader cells, creating tracks within the extracellular matrix by matrix remodeling (60). In our model, we could detect tumor cells’ enhanced aggressive behavior when co-cultured with stromal fibroblasts. In addition, the migration pattern of MDA-MB-231 cells was changed from a collective cell migration type to a single-cell migration type when they were co-cultured with fibroblasts. Recent studies showed that the induction of the epithelial-to-mesenchymal transition (EMT) process in cancer cells can be a spectrum of intermediary responses, ranging from complete EMT to partial response. EMT states’ variability corresponds to diverse effects on cell mobility and migration type (61, 62). The MDA-MB-232 cell line in our study could be not fully EMT-engaged (63). The co-cultured fibroblasts produce pro-migratory stimulus, including cytokines and growth factors (9). Also, the early invasion of fibroblasts into the collagen matrix gel could alter its composition and geometry (59). These changes could enhance the EMT state in co-cultured MDA-MB-231 cells. Increasing cell engagement in EMT further reduces cell-cell adhesion and lessens the apicobasal epithelial polarity. The complete resolution of cell-cell junctions and cell polarity favors cell individualization and eventually shifts the cell migration from collective to single-cell type (64).

As a proof-of-concept, we demonstrate the amenability of our model to be used as *in vitro* drug testing platform for assessing the metastatic response of MDA-MB-231 breast cancer cells to drug treatment. We screened a library of 86 targeted anti-cancer drugs with annotated molecular targets (**Supplementary Table 1**). The phenotypic drug screen revealed 30 compounds that reduce MDA-MB-231 breast cancer cell intravasation by more than 60% compared to controls. Of note; we detected two drugs: Olaparib and Talazoparib tosylate that are poly (ADP-ribose) polymerase (PARP) inhibitors, that prevent DNA damage repair in cancer cells. Olaparib and Talazoparib are approved clinically as monotherapies for triple-negative breast cancer with germline BRCA mutations (65). Further multi-parametric analysis that combined the findings from the metastasis-on-chip screen with those of tumor cell proliferation and 2D migration screens identified eight drug groups with distinct cellular responses (66, 67). Furthermore, we investigated the molecular targets of drugs enriched in each cluster (**Supplementary Table 2**). Many hormone drugs such as Tamoxifen were clustered in the cluster 2, the same cluster of negative control. This was expected as MDA-MB-231 cells are negative to hormonal receptors. MEK inhibitors were enriched in cluster 8, where tumor cell invasion and intravasation were strongly inhibited. The effect on tumor cell intravasation is likely a secondary effect on the cell invasion in this cluster. The role of the MEK signaling pathway in tumor cell invasion has been extensively reported in the literature (68, 69). Hence, the identification of MEK inhibitors in our screen was unsurprising but confirmative of the validity of the screening outcome. In preclinical studies, MEK inhibitors have shown anti-cancer activity in triple-negative breast cancer cell lines (70). In contrast, MDA-MB-231 cells were unresponsive to several EGFR and HER2 inhibitors, which were significantly enriched in clusters 1 & 3, reducing 3D cell invasion of ECM by less than 10% compared to controls. However, they reduced cell migration by 30% compared to controls in the 2D migration assay of tumor cell monoculture. Thus, these findings suggested that the cellular response to the drug in a 3D complex microenvironment could be different for some molecular targets to their cellular response in conventional 2D cell cultures (71), since 2D migration assay and cell invasion were positively correlated as whole data (**Supplementary Figure 6**). In clinical practice, Erlotinib, EGFR inhibitor, was efficient in patients with estrogen receptor-positive breast cancer; however, it had little effect on triple-negative breast cancer patients (72). Likewise, Afatinib, an EGFR inhibitor, did not show any significant benefit on the clinical outcome for HER2-positive breast cancer patients (73). There is no clinical data about Afatinib on HER2-negative breast cancer because the randomized clinical trial, TRIO-020, which was supposed to evaluate Afatinib efficacy on HER2-negative breast cancer, was discontinued (74, 75). MDA-MB-231 cells highly express EGFR, but they are HER2 negative (76). Therefore, it is expected that HER2 inhibitors like Lapatinib to be inefficient in MDA-MB-231 cells in our study. Nevertheless, we found that Neratinib, a HER2 inhibitor in the library, significantly reduced MDA-MB-231 cell invasion and intravasation. In clinical practice, both Lapatinib and Neratinib are FDA-approved drugs for treating the subtype of HER2-positive breast cancer patients. Lapatinib has a high affinity for EGFR and HER2 targets. In contrast, Neratinib also binds with high affinity to other targets: MEK1 and MEK2, which might explain its anti-metastatic effects in our assay (77). Previous reports showed that MDA-MB-231 cells with K753E mutation were resistant to Lapatinib but not Neratinib and clinically showed that the HER2 K753E mutation was enriched in metastatic lesions (78). Several VEGFR inhibitors, enriched in Cluster 7, demonstrated a reduction of MDA-MB-231 cell invasion, intravasation, and proliferation. It has previously been shown that vascular endothelial growth factor (VEGF) can enhance the proliferation of cancer cell lines, including MDA-MB-231, and pharmacological VEGFR inhibition reduces their proliferation. Several mechanisms were proposed to explain the effect of VEGFR inhibitors on cancer cell proliferation, such as increased mitochondrial biogenesis and ROS production (79). VEGF-A/NRP1 axis was suggested to confer cancer stem cell traits in MDA-MB-231 breast cancer cells by activating the Wnt/β-catenin pathway. Expression of VEGFR1 has been reported in MDA-MB-231, followed by the observation that tumor cell growth is supported by selective VEGFR1 signaling and it is mediated by downstream activation of MAPK/ERK and PI3K/Akt pathways (80, 81). Of note; VEGFR inhibitors showed significant cytotoxicity against the endothelial cell monolayer of the microvessel, disturbing the endothelial barrier integrity. VEGFR2 is a receptor tyrosine kinase stimulated when VEGF binds and primarily mediates several endothelial functions such as cell survival, migration, endothelial monolayer permeability, and angiogenesis (45). Our experiments’ long-term incubation with VEGFR inhibitors impaired endothelial functions and microvessel disintegration. VEGFR inhibitors are widely used as anti-angiogenic agents to treat several advanced metastatic cancers. However, strong anti-angiogenic therapy could destabilize the intra-tumoral microvessels, leading to tumor cell intravasation. Also, a substantial reduction of tumor microvasculature could increase hypoxia inside the tumors, promoting metastasis (82–84). Pre-clinical models of breast cancers showed that sunitinib enhanced lung and liver metastasis (85) due to destabilizing intra-tumoral microvessels. Taken together, the drug screen demonstrates that our metastasis-on-chip platform is suitable as pre-clinical drug testing and drug discovery platform, and it could be integrated with other established assays (e.g., tumor growth or motility assays) to enhance the drug profiling outcome.

We showed that Imatinib and Sorafenib inhibit MDA-MB-231 breast cancer cell intravasation without significantly affecting cell invasion or proliferation, suggesting that the drug mechanism of action is related to the interaction between tumor cells and endothelial cells. Both drugs did not disturb the endothelial barrier integrity of microvessels. Imatinib is a selective multi-kinase inhibitor that targets BCR-ABL, KIT and PDGFR and is an FDA-approved drug to treat chronic myeloid leukemia. Later its clinical use extended to other cancer types, such as Gastrointestinal stromal tumors (GISTs) (86–89). Imatinib prevents the proliferation of tumor cells that express BCR-ABL fusion proteins. Consistent with previous reports, we showed that Imatinib attenuates endothelial barrier dysfunction caused by the co-culture with MDA-MB-231 breast cancer cells/fibroblasts and decreased VEGF-induced endothelial permeability. It has been reported that Imatinib improves endothelial barrier dysfunction through ABL inhibition (90), and ABL kinases are required to induce endothelial permeability by VEGF and other factors (91). Indeed, the loss of ABL kinase activity was associated with increased endothelial barrier-stabilizing GTPases Rac1 and Rap1 activity and inhibition of actomyosin contractility (92). Another study showed that ABL1 Knockdown experiments by siRNA reduced actin remodeling and Paxillin phosphorylation leading to a modulation of endothelial cell migration (43). Our findings highlight that the effects on vascular barrier stability could be the underlying mechanism associated with Imatinib’s anti-metastatic effects.

To summarize, we have developed a high-throughput metastasis-on-chip-based assay that recapitulates the early stages of breast cancer metastasis, including tumor cell invasion and intravasation. Establishing automated imaging and analysis methods for this platform allowed us to conduct a phenotypic drug screen. The screen resulted in several drugs such as PARP inhibitors that modulate tumor cell invasion and intravasation, which further validated the efficacy of our model to use it as a platform to identify drugs with anti-metastatic activity. Our findings identified Imatinib as an effective drug for inhibiting tumor cell intravasation. However, further experiments are required to confirm this effect *in vivo*. In the current assay, we acknowledge that it would be more relevant to integrate breast tumor-associated cells (endothelial cells and fibroblasts) in our assay. However, HUVEC and skin fibroblasts were widely used as a useful model for research on human endothelium, fibrosis, and cancer biology. Of note, our current optimized assay would facilitate a straightforward future application of the tumor-associated cells. We also acknowledge that our assay does not include other essential cell components of tumor environment and intravasation process, such as immune cells and cancer-associated adipocytes. Further studies will be needed to optimize the integration of multiple cancer-associated cell types in the current assay.

## Data availability statement

The original contributions presented in the study are included in the article/**Supplementary Material**. Further inquiries can be directed to the corresponding author.

## Ethics statement

Ethical approval was not required for the studies on humans in accordance with the local legislation and institutional requirements because only commercially available established cell lines were used.

## Author contributions

LO: Data curation, Formal analysis, Investigation, Methodology, Writing – original draft. HF: Investigation, Methodology, Writing – review & editing. JE: Investigation, Methodology, Writing – review & editing. AA: Conceptualization, Data curation, Formal analysis, Funding acquisition, Investigation, Methodology, Project administration, Supervision, Writing – original draft, Writing – review & editing.

## Supplementary material

Supplementary Movie 1 3D reconstruction of metastasis-on-chip.

Supplementary Table 1 Normalized drug screen data with drug annotation.

Supplementary Table 2 Enrichment of drug molecular targets in the clusters.
