# Supplementary material for: Development of a cancer metastasis-on-chip assay for high throughput drug screening
Source: Front Oncol. 2024 Jan 4;13:1269376. doi: 10.3389/fonc.2023.1269376 (PMC10794518; doi:10.3389/fonc.2023.1269376)
Supplement: Supplementary file 1 [file DataSheet_1.pdf]

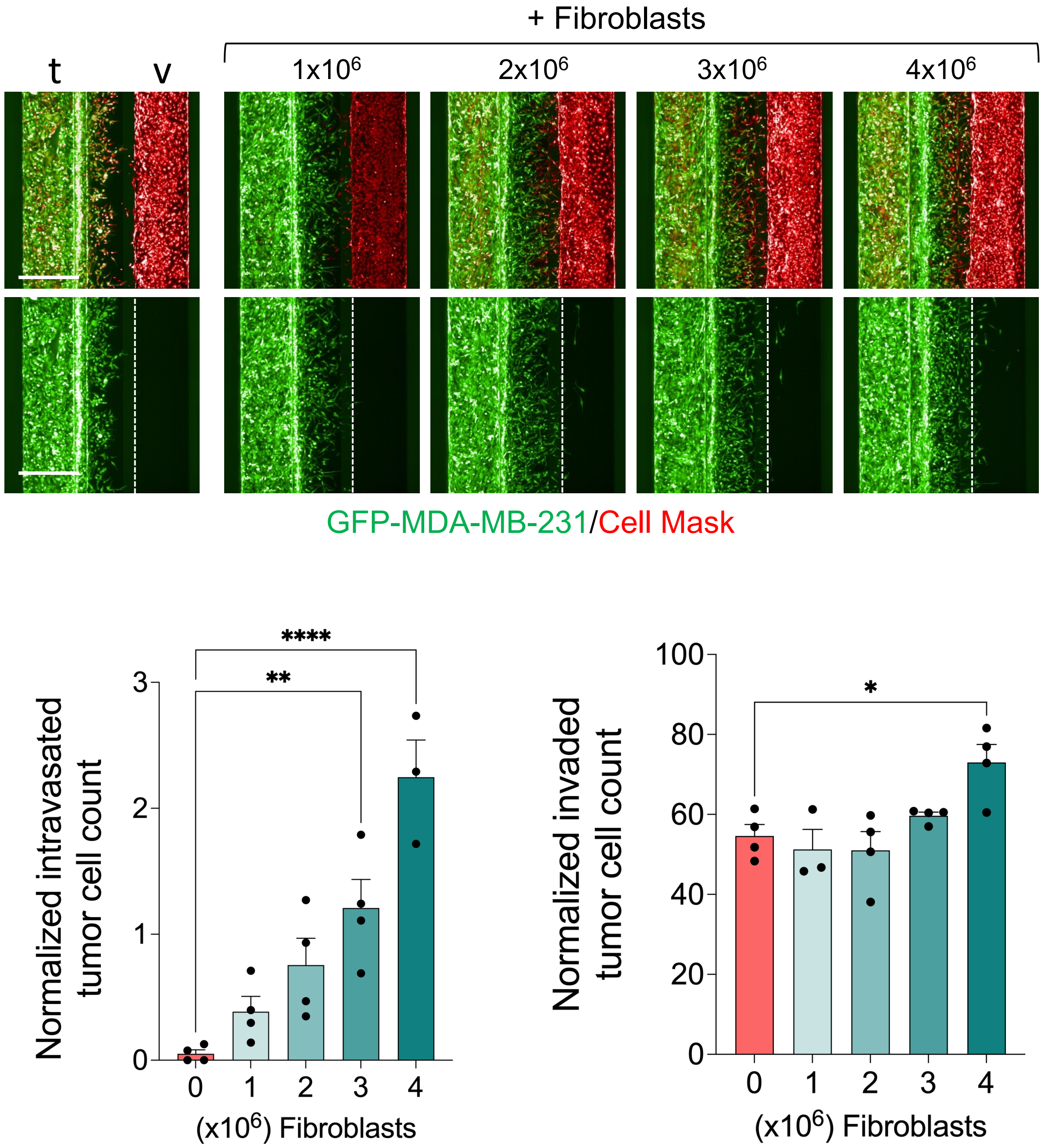

Supplementary Figure 1. Optimization of fibroblast number for the metastasis-on-chip model. After the microvessel (v) formation, GFP-labelled MDA-MB-231 cells were seeded alone at a density of $4x10^6$ cells per ml or mixed with three different concentrations of adult dermal fibroblasts ($1x10^6$, $2x10^6$, $3x10^6$ or $4x10^6$ cell per ml). Cell suspension mixture (3µl) was added into the inlet of the tumor channel (t). After five days, cells were fixed and stained with deep red cell mask (cell body, red). *Upper*, representative confocal z-stack images showing the different concentration of fibroblasts. Dotted line delimits the microvessel border. Scale bar= 500 µm. *Lower,* quantification of intravasated and invaded tumor cell number for each condition. The intravasated and invaded tumor cell count were normalized to the total tumor cell number in the tumor channel (t). Data are expressed as mean ± SEM; n=3-4 chips per condition; one-way ANOVA followed by Bonferroni post hoc test, *p<0.05, **p<0.01, and ****p<0.0001 compared to controls.

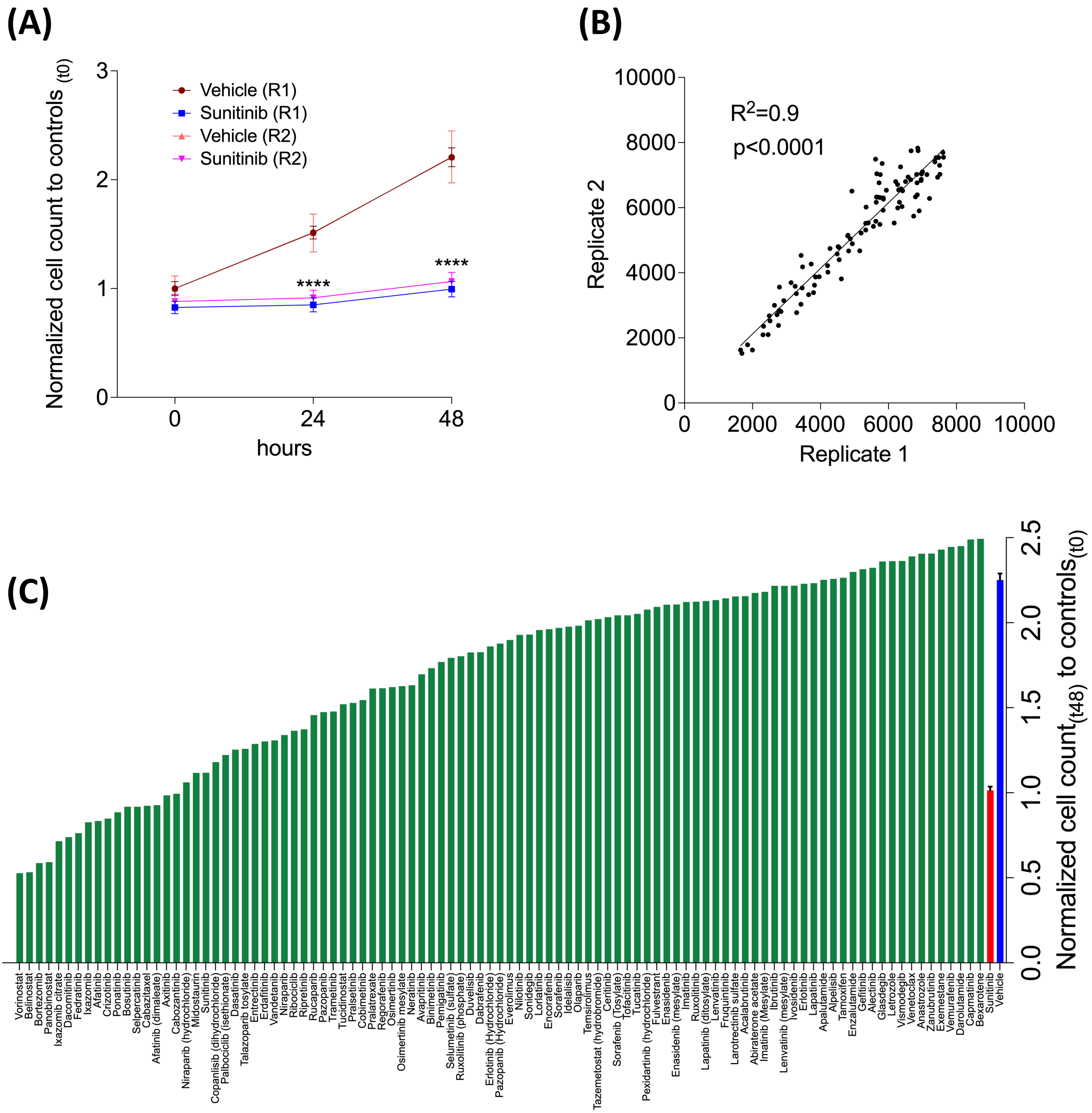

Supplementary Figure 2. High content screen of the targeted anti-cancer drug library to profile their effect on MDA-MB-231 tumor cell proliferation. GFP-labelled MDA-MB-231 were seeded at density of 5000 cells per well in 96-well plates. The targeted anti-cancer drug library (99 compounds) were incubated for 48 hours. The plate wells were imaged and cell number were quantified at three time points: 0h, 24h and 48h using high content imaging system (Operetta). The drug library was screened in duplicate at a final concentration of 5 µM. (**A**) Time course of cell proliferation rate for the negative controls (DMSO 0.05%) and positive controls (Sunitinib, 5 µM). Data are expressed as mean ± SEM; n=8 wells per replicate; one-way ANOVA followed by Bonferroni post hoc test, ****$p<0.0001$ compared to vehicle (0h). (**B**) Scatter plot showing the correlation of raw screen data between the two replicates measured in the screen at time point 48h. (**C**) Ordered bar chart distribution showing the effect of compound library on mean tumor cell proliferation rate as measured at 48h time point. The replicate data were averaged and then normalized to the controls (0h). Vehicle (n=16) and Sunitinib (n=16) data values were expressed as mean ± SEM.

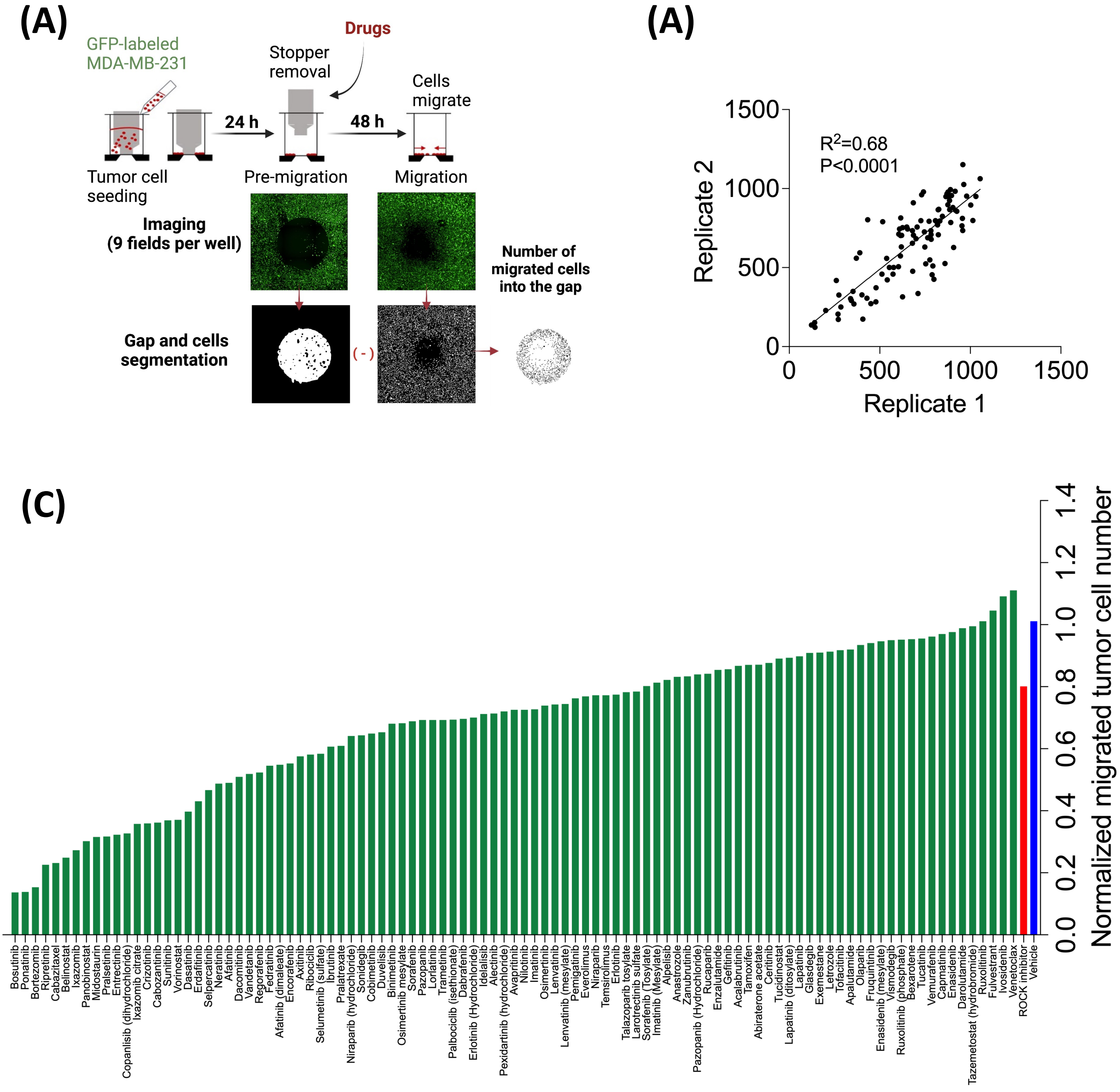

Supplementary Figure 3. High content screen of the targeted anti-cancer drug library to evaluate their effect on MDA-MB-231 cell migration. (**A**) Workflow illustrating the steps of drug screen using the wound healing 2D migration assay. The quantification of the migrated cell number into the gap was conducted using Harmony® image analysis software and NIH Image J. The drug library was screened in duplicate at a final concentration of 5 μM. (**B**) Scatter plot showing the correlation of raw screen data between the two replicates measured in the screen at time point 48h. (**C**) Ordered bar chart distribution showing the effect of compound library on mean tumor cell migration. The replicate data were averaged and then normalized to the controls. Vehicle (n=16) and ROCK inhibitor (GSK429286) (n=16) data values were expressed as mean ± SEM.

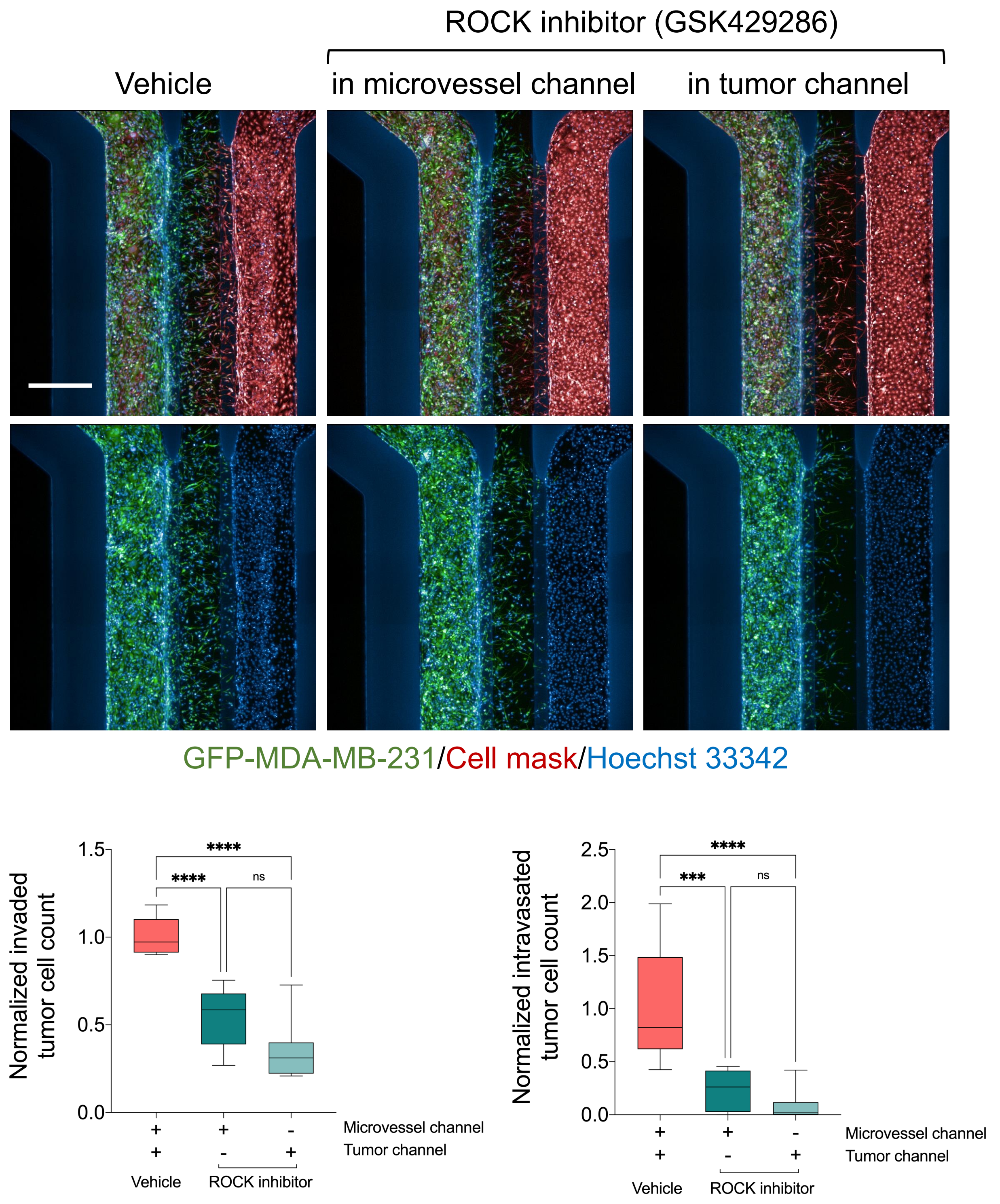

Supplementary Figure 4. Optimization of ROCK inhibitor as a positive control for the high content screening using metastasis-on-chip model. The ROCK inhibitor (GSK429286) was added either into the tumor channel or into microvessel perfusion channel. The drug was replenished once after 2 days. After day 6 of tumor cell/fibroblasts seeding, the chips were fixed and stained with deep red cell mask (cell body) and Hoechst 33342 (nuclei). *Upper*, representative confocal z-stack images of metastasis-on-chips treated with vehicle (DMSO) or GSK429286 (5 μm). Scale bar= 500 μm. *Lower,* quantification of the intravasated and invaded tumor cell number. Both parameters were normalized to the total tumor cell number in the tumor channel. Data are presented as box plots, with maximum, minimum, and quartile range. ; n=6-7 chips per condition; one-way ANOVA followed by Bonferroni post hoc test, $^{**}p<0.01$, $^{***}p<0.001$, and $^{****}p<0.0001$ compared to vehicle.

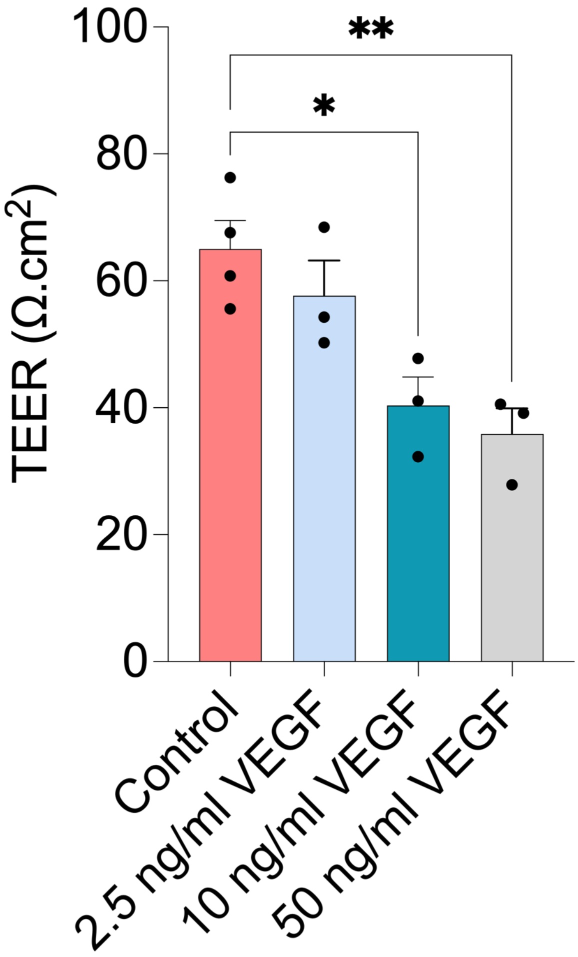

Supplementary Figure 5. Effect of VEGF on HUVEC monolayer permeability using TEER measurement. Data are expressed as mean ± SEM; one-way ANOVA followed by Bonferroni post hoc test, *p<0.05 and **p<0.01 compared to controls

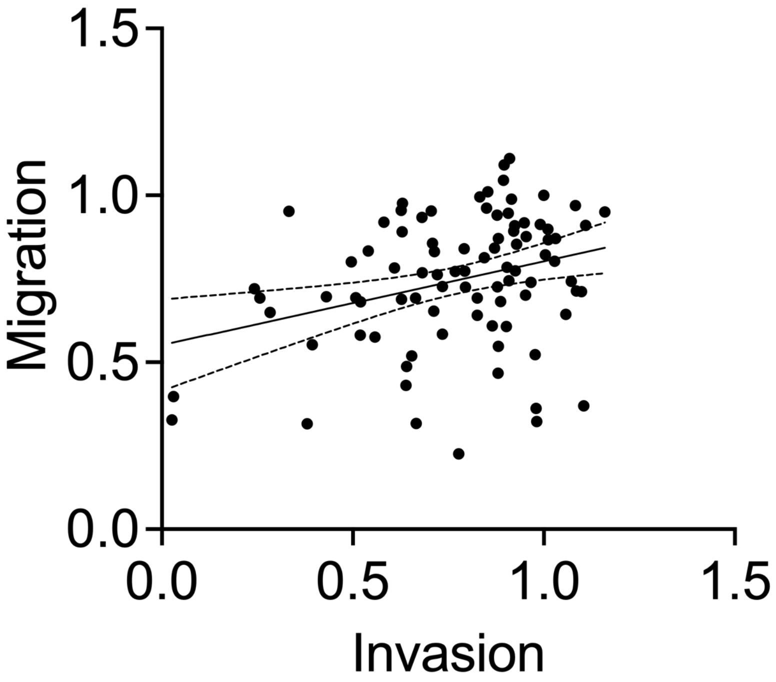

Supplementary Figure 6. Scatter plot showing the correlation between the invasion and 2D migration parameters resulted from the drug screens. Pearson correlation test, r=0.3, p=0.004.
